# Risk of endometrial polyps in women with endometriosis: a meta-analysis

**DOI:** 10.1186/s12958-015-0092-2

**Published:** 2015-09-17

**Authors:** Qiao-Mei Zheng, Hong-luan Mao, Yan-Jing Zhao, Jing Zhao, Xuan Wei, Pei-Shu Liu

**Affiliations:** Department of Obstetrics and Gynecology, Qilu Hospital of Shandong University, 107 Wenhua Xi Road, Jinan, Shandong 250012 People’s Republic of China; Department of Surgery, 92403 Military Hospital, 38 Luoxing Xi Road, Fuzhou, Fujian 350015 People’s Republic of China

**Keywords:** Endometriosis, Endometrial polyps, Meta-analysis

## Abstract

**Background:**

Endometrial polyps (EP) and endometriosis are both estrogen-dependent overgrowths of the endometrium. Several studies have shown a higher frequency of EP in endometriosis patients when compared with women without endometriosis. Therefore, we performed a meta-analysis to investigate the risk of EP in women with endometriosis.

**Methods:**

This meta-analysis searched for articles published between 1964 and 2014 in PubMed, Embase, and Cochrane Library, as well as in Chinese databases, including CNKI, VIP and Wanfang, regarding the association between endometriosis and EP. Nine cohort studies and one case–control study including 2896 women were included in this meta-analysis. The EP risk was evaluated using relative risk (RR) with a 95 % confidence interval (CI). Heterogeneity, small study effect and publication bias were assessed using Higgins I^2^, sensitivity analysis and funnel plots, respectively.

**Results:**

The risk of EP increased in women with endometriosis compared with those without endometriosis (the pooled RR, 2.81; 95 % CI, 2.48–3.18). No significant heterogeneity, small study effect or publication bias was found. The risk of EP slightly increased in women with endometriosis at stages 2–4 compared with those at stage 1 (Pooled effect size: stage 2 versus stage 1, RR, 1.22, 95 % CI, 1.04 - 1.42; stage 3 versus stage 1, RR, 1.23, 95 % CI, 1.06–1.42; stage 4 versus stage 1, RR, 1.29, 95 % CI, 1.11–1.51; stages 2–4 versus stage 1, RR, 1.24, 95 % CI, 1.10–1.40); however, no significantly different risk of EP in women with endometriosis existed between the other stages.

**Conclusion:**

The results suggest that it is important to identify whether patients with endometriosis also have EP and then remove any coexisting EP via hysteroscopy, especially for infertile patients. This process will be clinically helpful to treat endometriosis-related infertility in patients with endometriosis, especially for those with endometriosis that is more serious than stage 1.

**Electronic supplementary material:**

The online version of this article (doi:10.1186/s12958-015-0092-2) contains supplementary material, which is available to authorized users.

## Background

Endometriosis is characterized by the presence of endometrial glands and stroma at extrauterine locations and affects approximately 10 % of women of reproductive age, resulting in dysmenorrhea, infertility and decreased quality of life [1, 2]. Although its exact pathogenesis is unclear, endometriosis is considered to be an estrogen-dependent disease [3]. The expression patterns of estrogen receptors (ER) and aromatase are both altered in patients with endometriosis [4–6]. Furthermore, estrogen metabolism in the endometrium of patients with endometriosis is quite different from that in normal endometrium [3].

Endometrial polyps (EP), the localized hyperplastic growth of endometrial glands and stroma, can affect up to 25 % of women, especially infertile women [7]. Similar to endometriosis, EP is also believed to be an estrogen-dependent disease [8]. The concentrations of ER and progesterone receptors (PR) were found to be significantly higher in the endometrium from women with EP than in normal endometrium [9, 10]. Moreover, the aromatase level was remarkably higher in the endometrium from EP patients than in control endometrium [11]. Recent evidence showed that EP was a potential cause for unexplained infertility and that polypectomy significantly increased the pregnancy rate [12].

Because of their common pathogenesis, endometriosis and EP may be associated with each other in some aspects. Emerging evidence suggests a higher frequency of EP in endometriosis patients compared with women without endometriosis [12–15]; however, whether endometriosis patients have a high risk of EP has not been demonstrated. The present article evaluated the association of endometriosis and EP. To investigate whether endometriosis patients have a high risk of EP, we performed a meta-analysis using previously published studies.

## Methods

### Search strategy and selection criteria

For the meta-analysis, we systematically and carefully searched PubMed, Embase, Cochrane Library, as well as Chinese databases, including CNKI (China National Knowledge Infrastructure), VIP (Chinese Scientific Journals Database) and Wanfang for relevant studies available online and published between 1964 and 2014. The search terms “endometriosis and endometrial polyps” or “endometriosis and uterine polyps” or “endometriosis and endometrial neoplasm” were used as key words. The searches were conducted independently by two authors (QM Zheng and YJ Zhao).

The included studies had to meet the following criteria: 1) endometriosis and EP diagnosed by surgery or pathology, 2) clearly described selection of controls, 3) human study, and 4) cohort or case–control study. Articles were excluded if they met the following criteria: 1) case report or review article, 2) animal or cell experiment, or 3) not published in English or Chinese.

### Selection of articles and data extraction

Two authors (QM Zheng and YJ Zhao) independently scanned the titles and abstracts and evaluated the potential eligibility of all of the studies according to the inclusion and exclusion criteria. Any disagreement in the process was resolved by discussion or the third author (J Zhao). The references of the selected articles were also checked for possible studies to include. Data extraction was also performed by the two authors using a standardized data-collection form. The following data were extracted from the eligible studies: the first author, year of publication, study population, study design, numbers of women with and without endometriosis, age, diagnosis of endometriosis and EP, numbers of patients with EP in women with and without endometriosis, numbers of endometriosis patients at r-AFS (revised-American Fertility Society) or AFS (American Fertility Society) stages 1–4, and numbers of women with EP at each stage of endometriosis. For articles lacking the relevant data, an E-mail was sent to the authors to request the information.

### Quality assessment

The quality of each study was independently assessed by the two authors using the Newcastle-Ottawa Scale (NOS), a validated tool for assessing the quality of non-randomized studies in meta-analyses [16]. The NOS uses a star system based on three aspects: selection of the study groups (up to four points), comparability of the groups (up to two points), and exposure or outcome (up to three points) for case–control or cohort studies. We included studies with an NOS score ≥ 5 in the current systematic review and meta-analysis.

### Statistical analysis

We primarily assessed the risk of EP in women with endometriosis compared with those without endometriosis. Further analysis was conducted to evaluate the risk of EP in women with endometriosis according to the stage of endometriosis. The meta-analysis was performed with STATA software (version 12.0 StataCorp, College Station, TX). All of the results for the binary outcomes are shown as the relative risk (RR) with a 95 % confidence interval (CI) in the case–control or cohort studies. The impact of heterogeneity was assessed using Higgins I^2^ [17]. When *P* > 0.1 or I^2^ ≤ 50 %, the fixed effect model using the Mantel-Haensze l method was used because it indicated acceptable heterogeneity. Conversely, the random effects model was used when *P* <0.1 or I^2^ > 50 %.

To establish the robustness of our results, a sensitivity analysis was performed by recalculating the pooled RR after deleting the studies one at a time. To identify the presence of publication bias, a funnel plot was produced using the logRR of each study on the X-axis and the standard error of the logRR of each study on the Y-axis. Furthermore, the Harbord test was used to test the symmetry of the funnel plot. *P* > 0.5 suggested that the funnel plot was symmetric and that no significant publication bias existed.

## Results

### Included studies and quality assessment

**Fig. 1:**
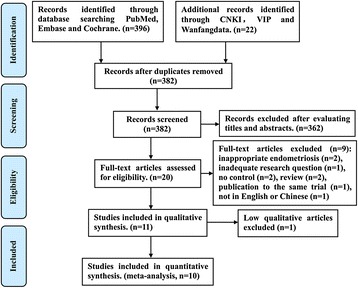
A flowchart depicting the selection of studies for the meta-analysis

As shown in Fig. 1, we collected 418 articles from the databases. After deleting the duplicates and screening titles and abstracts, 20 articles remained for eligibility assessment. Of the 20 studies selected after searching the database, only 11 studies were found to meet the inclusion criteria. The 11 studies, including 10 cohort studies and 1 case–control study, were published between 1996 and 2014. Of the 11 studies, one was excluded for its NOS score < 5. Therefore, 9 cohort studies [12, 13, 15, 18–23] and 1 case–control study [24] including 1286 women with endometriosis and 1610 women without endometriosis were included in the meta-analysis (Table 1). The studies were conducted in the United States (*n* = 1), Korea (*n* = 2), and China (*n* = 7). The NOS scores of the 10 included studies ranged from 5 to 7 (Additional file 1: Table S1), and the mean score was 6.1 (± SEM 0.2769). Of the 10 studies, only 5 studies analyzed the association of EP in women with endometriosis according to AFS or r-AFS stages. The NOS scores of the 5 studies ranged from 6 to 7 (Additional file 2: Table S2), and the mean score was 6.8 (± SEM 0.20).

**Table 1:** Summary of included studies

Author, year, place	Study population	Study design	Cases	Controls	Parameter measured	Result
McBean, 1996, USA	Women who had a HSG, hysteroscopy, and laparoscopy	Case–control	32 women with hysterosalpingograms proved polyps or polypoid endometrium	88 women with hysterosalpingograms proved absence of polyps or polypoid endometrium	Number of patients with endometriosis	Cases: 27; controls: 19
Mi Ran Kim, 2003, Korea	Infertile women undergoing laparoscopy	Cohort	92 (mean age, 30.6 ± 3.9 years) had laparoscopy-proved endometriosis	91 (mean age, 30.8 ± 4.6 years) had laparoscopy-proved absence of endometriosis	Number (%) of patients with endometrial polyps	Cases: 43 (46.7 %); controls: 15 (16.5 %)
Shuyun Zhao, 2006, China	Infertile women undergoing laparoscopy or laparotomy	Cohort	94 (mean age, 30.6 ± 4.07 years) had laparoscopy-proved endometriosis	98 (mean age, 30.8 ± 3.4 years) had laparoscopy-proved chronic pelvic inflammation; 116 (mean age, 30.7 ± 2.61 years) had laparoscopy-proved normal pelvic	Number (%) of patients with endometrial polyps	Cases: 20 (21.3 %); chronic pelvic inflammation: 4 (4.1 %); normal pelvic: 4 (3.4 %)
Jae Sun Park, 2009, Korea	Infertile women undergoing laparoscopy	Cohort	434 (mean age, 31.38 ± 4.37 years) had laparoscopy-proved endometriosis	197 (mean age, 31.05 ± 5.48 years) had laparoscopy-proved the absence of endometriosis	Number (%) of patients with endometrial polyps	Cases: 274 (63.0 %); controls: 58 (29.8 %)
Licong Shen, 2011, China	Infertile women undergoing simultaneous laparoscopy and hysteroscopy	Cohort	158 (mean age, 31.0 ± 4.2 years) had laparoscopy-proved endometriosis	273 (mean age, 30.7 ± 5.3 years) had no endometriotic foci found during laparoscopy	Number (%) of patients with endometrial polyps	Cases: 108 (68.35 %); controls: 56 (20.51 %)
Yubin Li, 2011, China	Infertile women aged 21–42 years undergoing laparoscopy and hysteroscopy	Cohort	84 (mean age, 30.87 ± 4.5 years) had laparoscopy-proved endometriosis	160 (mean age, 30.87 ± 4.5 years) had laparoscopy-proved the absence of endometriosis	Number (%) of patients with endometrial polyps	Cases: 16 (19.0 %); controls: 12 (7.5 %)
Hong Jin, 2013, China	Women undergoing surgery or physical examination	Cohort	150 aged 23–53 (mean age, 41.8 years) had surgery and pathology proved endometriosis	158 aged 23–53 (mean age, 39.11 years) had ultrasound proved the absence of endometriosis	Number (%) of patients with endometrial polyps	Cases: 23 (15.33 %); controls: 10 (6.33 %)
Fuqin Li, 2013, China	Infertile women aged 20–42 years undergoing laparoscopy and hysteroscopy	Cohort	42 (mean age, 28.5 ± 2.5 years) had laparoscopy-proved endometriosis	78 (mean age, 28.5 ± 2.5 years) had laparoscopy-proved the absence of endometriosis	Number (%) of patients with endometrial polyps	Cases: 8 (19.0 %); controls: 4 (5.1 %)
Yanru Li, 2013, China	Infertile women, aged 20–42 years undergoing laparoscopy and hysteroscopy	Cohort	81 (mean age, 29.5 ± 4.71 years) had laparoscopy-proved endometriosis	110 (mean age, 29.5 ± 4.71 years) had laparoscopy-proved the absence of endometriosis	Number (%) of patients with endometrial polyps	Cases: 17 (21 %); controls: 8 (7.3 %)
Gaixiang Xu, 2014, China	Infertile women undergoing simultaneous laparoscopy and hysteroscopy	Cohort	119 (mean age, 31.1 ± 3.0 years) had laparoscopy-proved endometriosis	241 (mean age, 30.7 ± 4.1 years) had laparoscopy-proved the absence of endometriosis	Number (%) of patients with endometrial polyps	Cases: 77 (64.7 %); controls: 51 (21.16 %)

### Outcomes

**Fig. 2:**
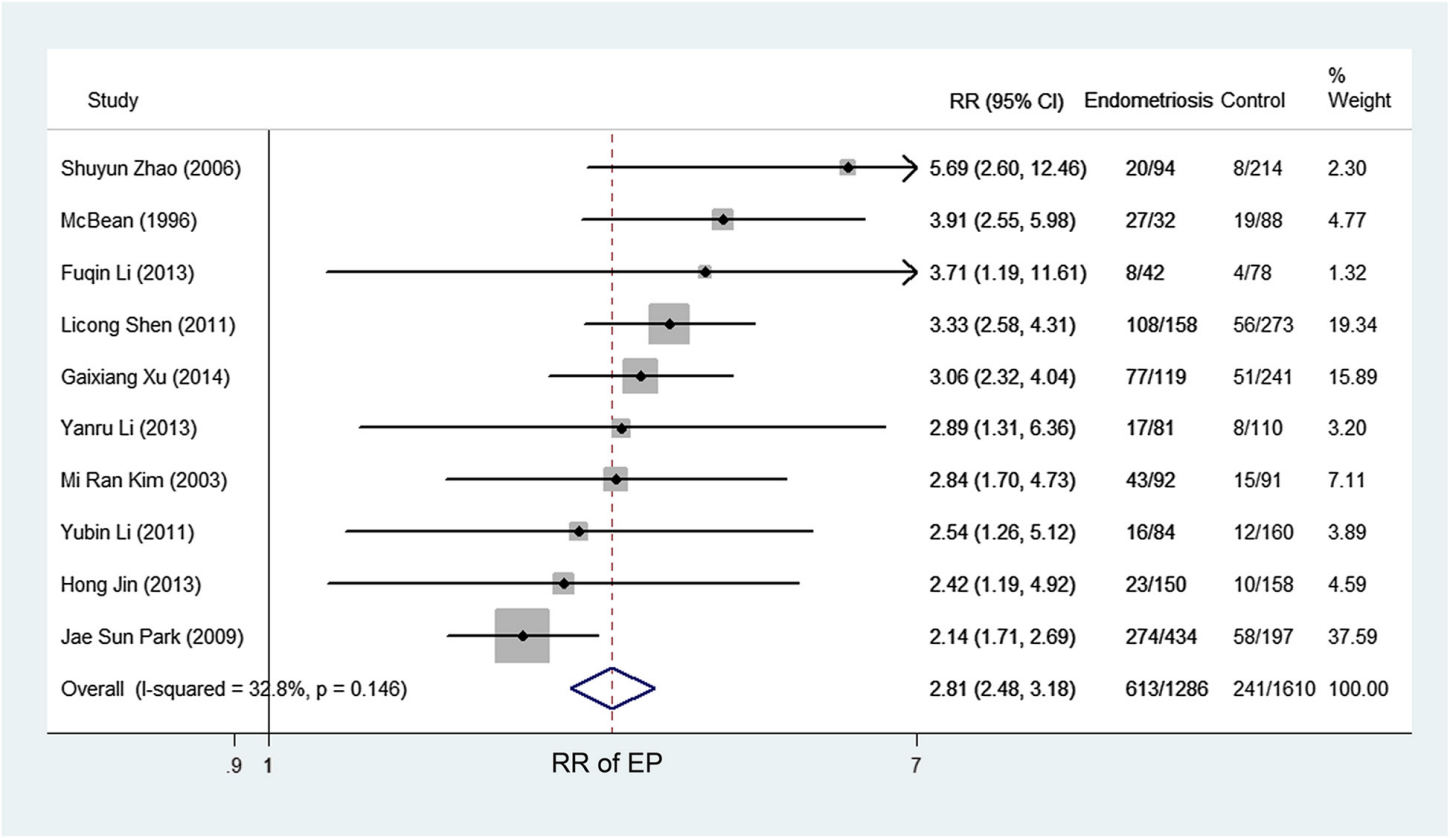
Forest plot of the 10 included studies evaluating the association between endometriosis and EP

**Fig. 3:**
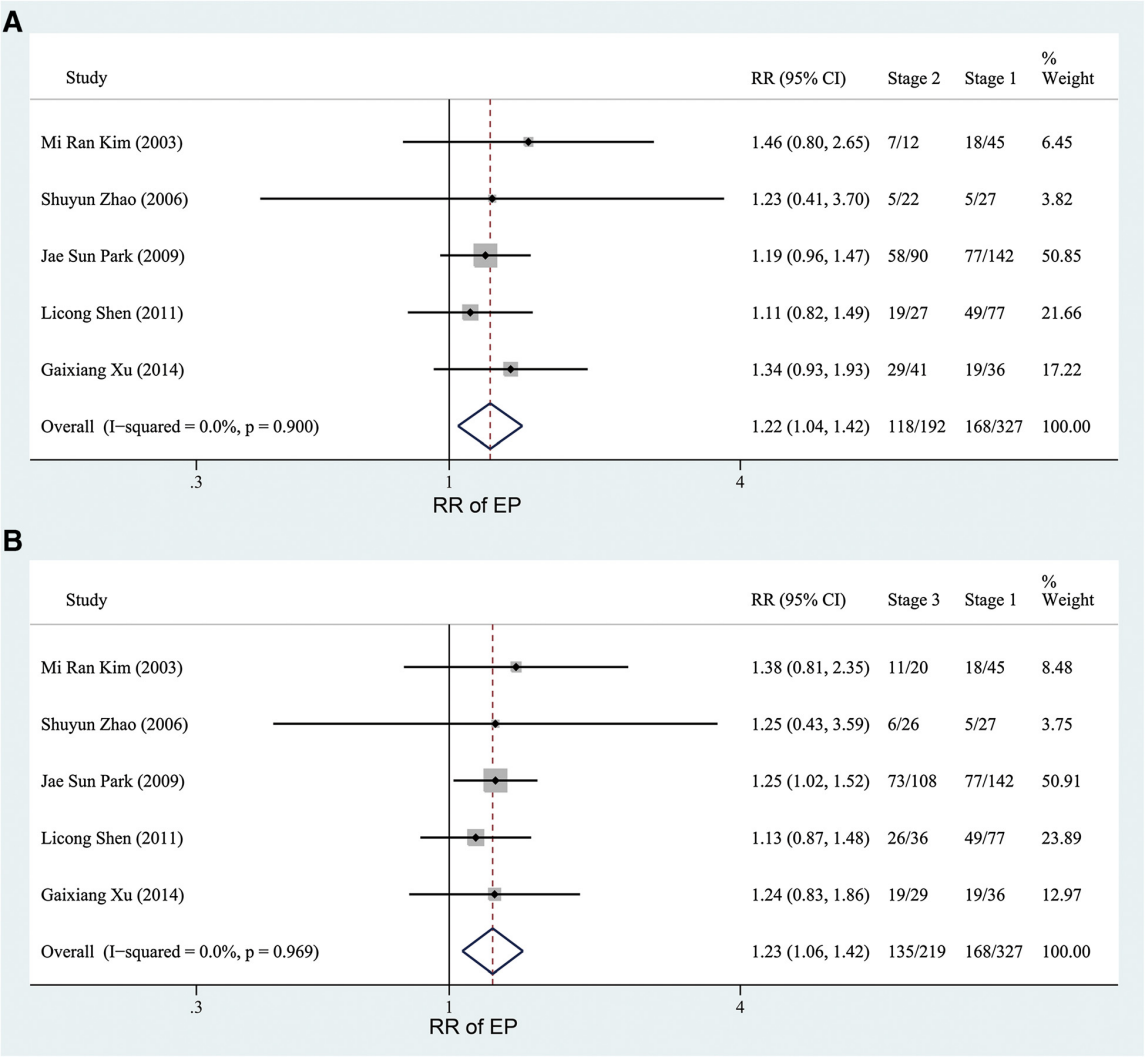
Forest plots of the 5 studies evaluating the association between EP and endometriosis according to the endometriosis stage (stages 2 and 3 versus stage 1)

**Fig. 4:**
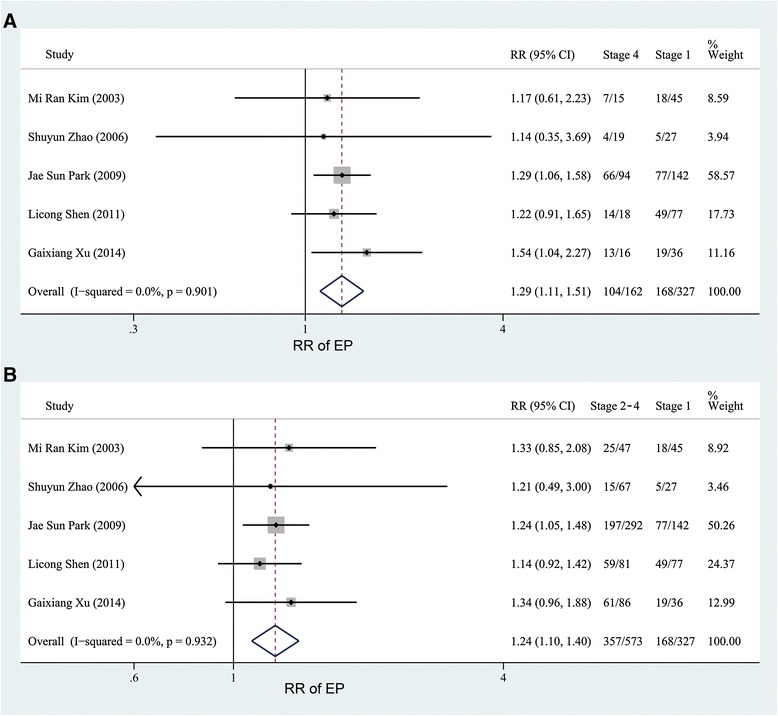
Forest plots of the 5 studies evaluating the association between EP and endometriosis according to the endometriosis stage (stages 4 and 2–4 versus stage 1)

The fixed effect model with the Mantel-Haenszel method was used because no obvious heterogeneity was found (*P* = 0.146, I^2^ = 32.8 %, Fig. 2). As depicted in Fig. 2, the pooled RR of 2.81 (95 % CI, 2.48–3.18) revealed that the risk of EP increased in women with endometriosis (47.67 %) compared with those without endometriosis (14.97 %). Then, we analyzed the risk of EP in women with different stages of endometriosis. For the articles reporting the association of EP in women with endometriosis according to AFS or r-AFS stages, the results of the heterogeneity tests were *P* >0.1, I^2^ = 0 % (Figs. 3 and 4, Additional file 3: Figure S1 and Additional file 4: Figure S2). Therefore, the fixed effect model with the Mantel-Haenszel method was used to calculate the pooled RR. As shown in Figs. 3 and 4, the risk of EP slightly increased in women with endometriosis at stages 2–4 (61.46, 61.64 and 64.20 %) when compared with those at stage 1 (51.38 %. Pooled effect size: stage 2 versus stage 1, RR, 1.22, 95 % CI, 1.04–1.42; stage 3 versus stage 1, RR, 1.23, 95 % CI, 1.06–1.42; stage 4 versus stage 1, RR, 1.29, 95 % CI, 1.11–1.51; stages 2–4 versus stage 1, RR, 1.24, 95 % CI, 1.10–1.40). However, no significantly different risk of EP in women with endometriosis existed between the other stages (Additional file 3: Figure S1 and Additional file 4: Figure S2).

### Publication bias and sensitivity analysis

**Fig. 5:**
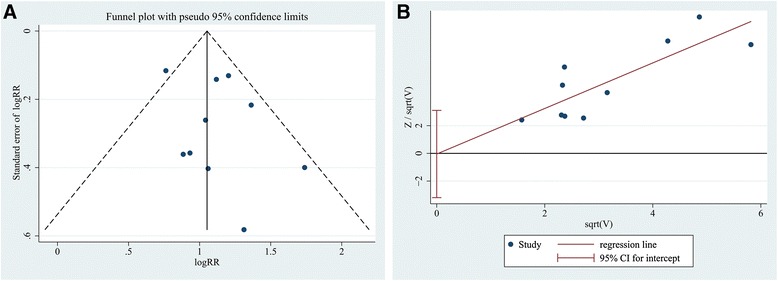
**a** Funnel plot, using data from the 10 studies evaluating the association between endometriosis and EP, with the logRR of each study on the X-axis and the standard error of the logRR on the Y-axis. **b** Harbord plot testing the symmetry of the funnel plot

**Fig. 6:**
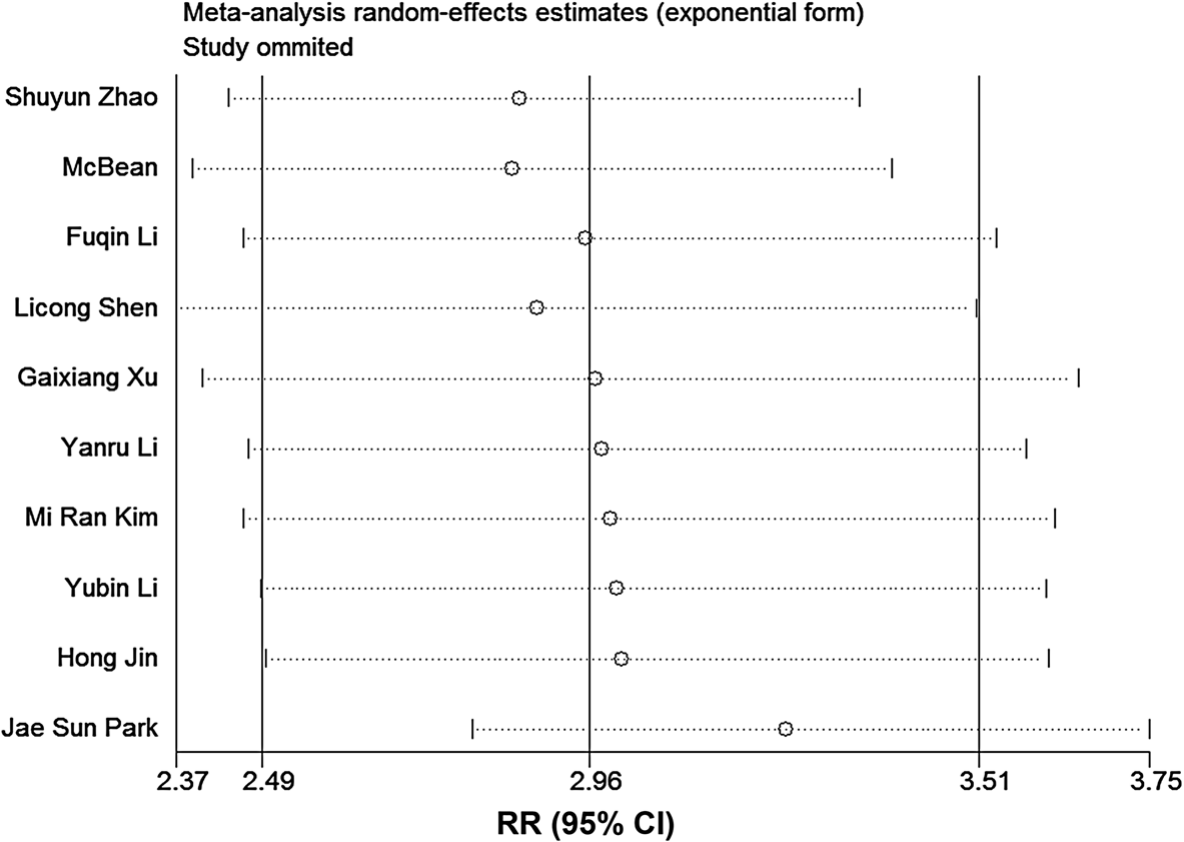
Sensitivity analysis of the meta-analysis

As indicated in Fig. 5, the funnel plot was visually symmetric. Then, the Harbord plot was used to test the symmetry of the funnel plot. The funnel plot was considered symmetric because the regression line had a positive slope through the origin of the coordinates (*P* = 0.973). Therefore, no obvious publication bias existed in the current meta-analysis. A sensitivity analysis was performed to evaluate whether any small study effect influenced the pooled effect size. No significant changes were observed in the leave-one-out sensitivity analysis (Fig. 6). Thus, the outcome of the current meta-analysis can be considered to be stable.

## Discussion

In this meta-analysis of cohort and case–control studies evaluating the association between EP and endometriosis, we found a significantly increased risk of EP in women with endometriosis compared with those without endometriosis. Moreover, the risk of EP in women with endometriosis slightly increased at stages 2–4 compared with those at stage 1; however, no significant difference was found for the risk of EP in women with endometriosis at stage 2, stage 3 or stage 4. Therefore, endometriosis patients have a significantly high risk of EP, especially for endometriosis that is more serious than stage 1.

The precise pathogenesis of endometriosis or EP is not clear. The most widely accepted theory is that the two diseases are both estrogen dependent [3, 8]. EP is the localized hyperplastic growth of endometrial glands and stroma, while endometriosis is the ectopic growth of endometrial glands and stroma. Both diseases include the overgrowth of the endometrium, a process that requires the support of estrogen. The expression patterns of ER and aromatase are both altered in endometriosis and EP patients [4, 6, 9, 11]. As an enzyme, aromatase can catalyze the conversion of androgen to estrogen, increasing the local concentration of estrogen. Estrogen metabolism, including aromatase and 17β-hydroxysteroid dehydrogenase type 2 (17βHSD2, an enzyme that inactivates estradiol to estrone), is altered in the eutopic endometrium of women with endometriosis compared with the eutopic endometrium of women without endometriosis [3]. Additionally, increased proliferation and decreased apoptosis have been observed in the eutopic endometrium of women with endometriosis compared with women without endometriosis [15, 25]. As a regulator of apoptosis, Bcl-2 was shown to be significantly increased in the eutopic endometrium of women with endometriosis, leading to the inhibition of apoptosis in the endometrium [26]. Herein, the eutopic endometrium in women with endometriosis was found to be different from that of women without endometriosis. Increased proliferation, decreased apoptosis and altered estrogen metabolism in the endometrium of women with endometriosis facilitated the presence of EP.

McBean first reported the association of endometriosis and EP in 1996 [24]. Since then, several studies have shown a higher frequency of EP in endometriosis patients than in women without endometriosis [12–15]. In accordance with previous studies, the present meta-analysis confirmed a significantly high risk of EP in women with endometriosis. Furthermore, the risk of EP was increased in endometriosis patients at stages 2–4 when compared with those at stage 1. No significant heterogeneity, small study effect or publication bias was found in the meta-analysis.

Although endometriosis patients were statistically shown to have a high risk of EP, this result should be carefully comprehended for the following reasons. First, only studies published in English and Chinese were included in this meta-analysis, and thus, studies published in other languages were omitted. Second, the pooled RR of the current meta-analysis may not be accurate because only a small number of studies were included. Third, the risk of EP in the endometriosis patients according to the endometriosis stage needs to be further ascertained because the included studies did not use consistent staging criteria for endometriosis. Consequently, this meta-analysis should be updated if studies addressing the association of endometriosis and EP are published in the future.

## Conclusion

The results suggest that it is important to determine whether patients with endometriosis also have EP and then remove any coexisting EP via hysteroscopy, especially for infertile patients. This procedure will be clinically helpful to treat endometriosis-related infertility in patients with endometriosis, especially for those with endometriosis that is more serious than stage 1.

## Additional files

Additional file 1:
**Table S1.** Quality of included studies using Newcastle-Ottawa scale. (DOC 38 kb) Additional file 2:
**Table S2.** Quality of studies analyzed the association of EP and endomeriosis stages using Newcastle-Ottawa scale. (DOC 30 kb) Additional file 3:
**Figure S1.** Forest plots of the 5 studies evaluating the association between EP and endometriosis according to the endometriosis stage (stages 3 and 4 versus stage 2). (TIFF 2120 kb) Additional file 4:
**Figure S2.** Forest plots of the 5 studies evaluating the association between EP and endometriosis according to the endometriosis stage (stage 4 versus stage 1, and stages 3–4 versus stages 1–2). (TIFF 2134 kb)

## Abbreviations

EP: Endometrial polyps
ER: Estrogen receptors
PR: Progesterone receptors
CNKI: China National Knowledge Infrastructure
VIP: Chinese Scientific Journals Database
r-AFS: Revised-American Fertility Society
AFS: American Fertility Society
NOS: The Newcastle-Ottawa Scale
RR: Relative risk
CI: Confidence interval
SEM: Standard error of the mean
17βHSD2: 17β-hydroxysteroid dehydrogenase type 2
