# Supplementary material for: Risk of endometrial polyps in women with endometriosis: a meta-analysis
Source: Reprod Biol Endocrinol. 2015 Sep 17;13:103. doi: 10.1186/s12958-015-0092-2 (PMC4574029; doi:10.1186/s12958-015-0092-2)
Supplement: Additional file 1: — Table S1. Quality of included studies using Newcastle-Ottawa scale. (DOC 38 kb) [file 12958_2015_92_MOESM1_ESM.doc]

**Supplementary Table 1**

Quality of included studies using Newcastle-Ottawa scale.

| **Author** | **Selection** | **Comparability** | **Outcome** | **Score** |
| --- | --- | --- | --- | --- |
| McBean, 1996 | * * * | * | * * | 6 |
| Mi Ran Kim, 2003 | * * | * * | * * * | 7 |
| Shuyun Zhao, 2006 | * * * | * * | * * | 7 |
| Jae Sun Park, 2009 | * * * | * * | * * | 7 |
| Licong Shen, 2011 | * * * | * | * * | 6 |
| Yubin Li, 2011 | * * * |  | * * | 5 |
| Hong Jin, 2013 | * * * |  | * * | 5 |
| Fuqin Li, 2013 | * * * |  | * * | 5 |
| Yanru Li, 2013 | * * * | * | * * | 6 |
| Gaixiang Xu, 2014 | * * * | * * | * * | 7 |
