# Supplementary figures and images for: Risk of endometrial polyps in women with endometriosis: a meta-analysis
Source: Reprod Biol Endocrinol. 2015 Sep 17;13:103. doi: 10.1186/s12958-015-0092-2 (PMC4574029; doi:10.1186/s12958-015-0092-2)

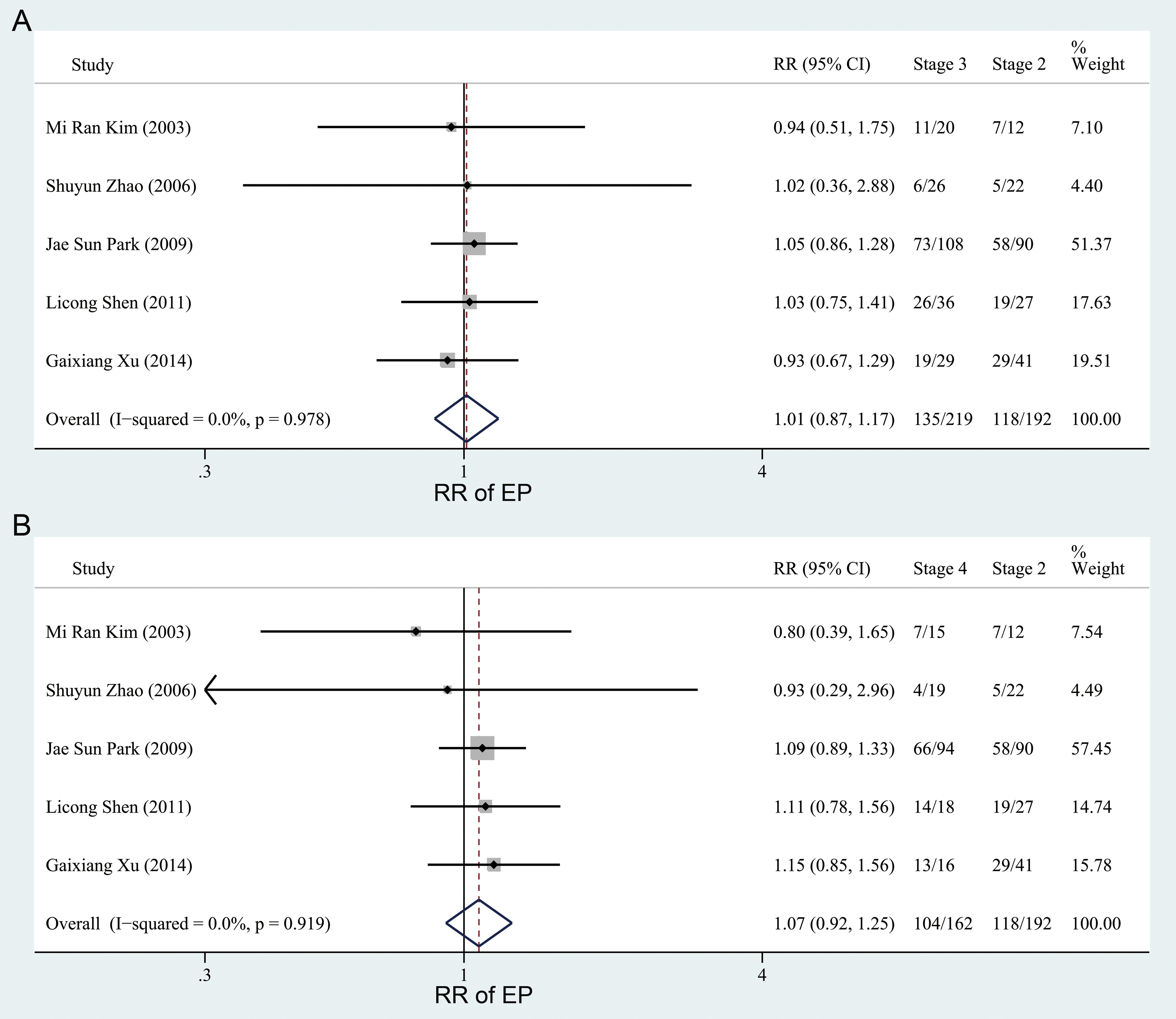

Supplement: Additional file 3: — Figure S1. Forest plots of the 5 studies evaluating the association between EP and endometriosis according to the endometriosis stage (stages 3 and 4 versus stage 2). (TIFF 2120 kb) [file 12958_2015_92_MOESM3_ESM.tif]

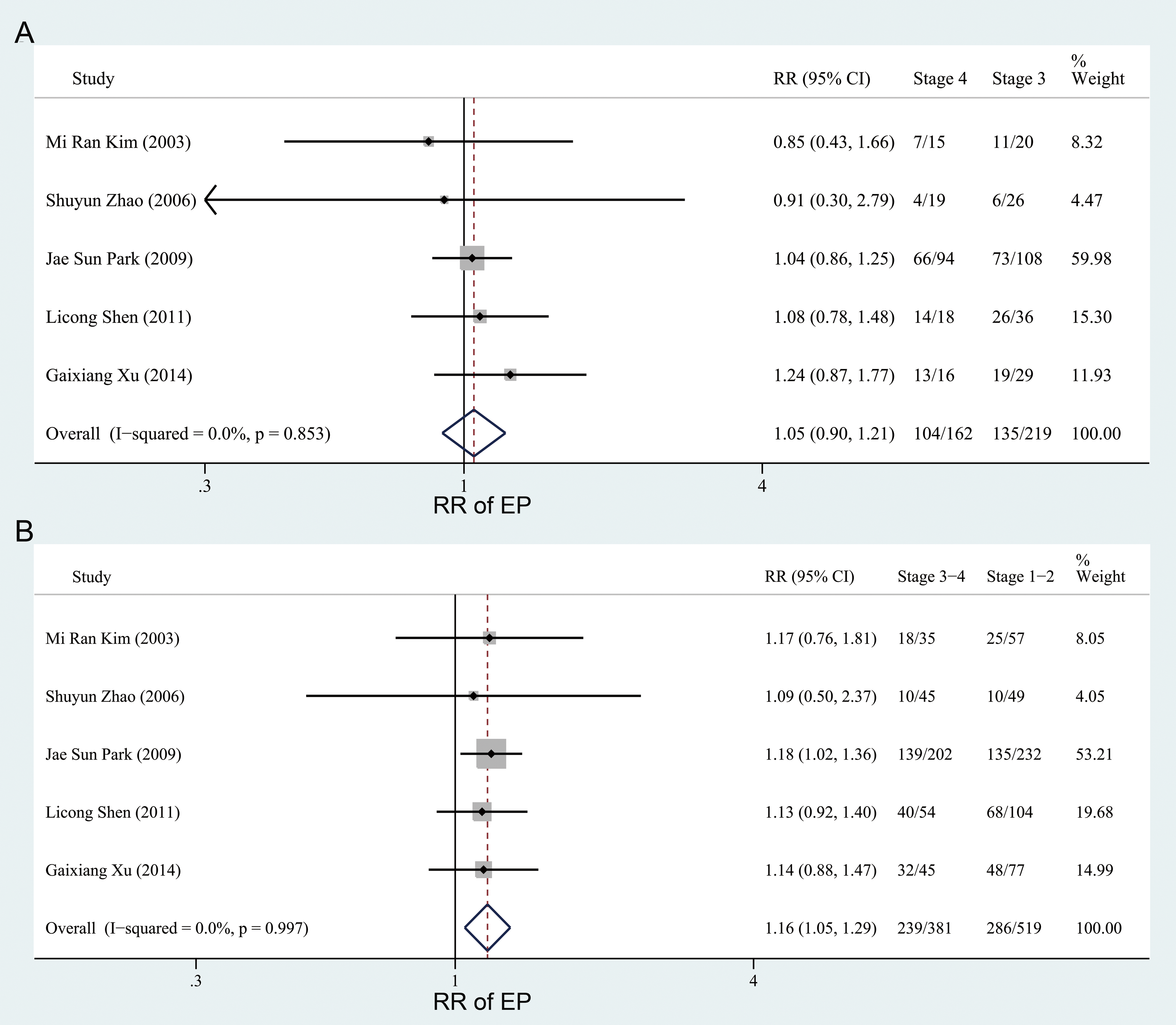

Supplement: Additional file 4: — Figure S2. Forest plots of the 5 studies evaluating the association between EP and endometriosis according to the endometriosis stage (stage 4 versus stage 1, and stages 3–4 versus stages 1–2). (TIFF 2134 kb) [file 12958_2015_92_MOESM4_ESM.tif]
